# Exploratory analysis of lateral pelvic sentinel lymph node status for optimal management of laparoscopic lateral lymph node dissection in advanced lower rectal cancer without suspected lateral lymph node metastasis

**DOI:** 10.1186/s12885-021-08480-6

**Published:** 2021-08-11

**Authors:** Masayoshi Yasui, Masayuki Ohue, Shingo Noura, Norikatsu Miyoshi, Yusuke Takahashi, Chu Matsuda, Junichi Nishimura, Naotsugu Haraguchi, Hajime Ushigome, Nozomu Nakai, Shiki Fujino, Keijiro Sugimura, Hiroshi Wada, Hidenori Takahashi, Takeshi Omori, Hiroshi Miyata

**Affiliations:** 1 Department of Gastroenterological Surgery, Osaka International Cancer Institute, Otemae 3-1-69, Chuo-ku, Osaka City, Osaka, Japan; 2 Department of Surgery, Toyonaka Municipal Hospital, Osaka, Japan; 3 Department of Gastroenterological Surgery, Osaka University, Osaka, Japan; 4 Department of Surgery, Osaka National Hospital, Osaka, Japan

**Keywords:** Rectal cancer, Sentinel lymph node, Lateral lymph node dissection, Laparoscopic surgery

## Abstract

**Background:**

Total mesorectal excision (TME) and lateral lymph node dissection (LLND) without radiotherapy (RT) are standard treatment for lower cT3/4 rectal cancers in Eastern countries. In comparative studies, both TME + LLND and RT + TME yield good local control. Although Japanese guidelines recommend LLND for locally advanced rectal cancers below the peritoneal reflection, LLND dissection of clinically negative lateral pelvic lymph nodes (LPLN) is controversial, and laparoscopic TME + LLND is technically challenging and time-consuming. New optical instruments for laparoscopy allow easy perioperative sentinel lymph node (SLN) identification using ICG. The SLN concept may facilitate accurate diagnosis of LPLN involvement, and thus reduce LLND in laparoscopic rectal cancer surgery. Here we investigated lateral pelvic SLN navigation surgery for SLN detection during laparoscopic rectal cancer surgery.

**Methods:**

This study included 21 patients with clinical StageII/III lower rectal cancer without LPLN enlargement, who underwent curative laparoscopic surgery. All patients underwent TME, followed by lateral SLN identification and biopsy using ICG, and then laparoscopic LLND. ICG fluorescence imaging was conducted using the laparoscopic near-infrared camera system.

**Results:**

Lateral SLNs were successfully identified in 16 (76.2%) of the 21 patients. Among the 15 patients without SLN tumor metastasis, the dissected lateral non-SLNs were all negative.

**Conclusions:**

A lack of metastasis in the lateral pelvic SLN seems to reflect a lack of metastases to all lateral LNs. Our present results suggest that this laparoscopic ICG-guided SLN strategy may be a low-risk and time-saving method to prevent laparoscopic LLND in cases with negative lateral pelvic lymph nodes.

**Supplementary Information:**

The online version contains supplementary material available at 10.1186/s12885-021-08480-6.

## Background

Total mesorectal excision (TME) is the international standard surgical procedure for lower rectal cancer. Anatomical studies have revealed that advanced tumors below the peritoneal reflection have a greater risk of spreading to lateral nodes [1–4]. Treatment of lymph node metastasis in the lateral pelvis has developed differently in Eastern versus Western countries. In the West, TME is commonly combined with neoadjuvant radiotherapy (RT) or chemoradiotherapy (CRT) treatment. On the other hand, in the East (principally in Japan), the standard treatment for lower cT3/4 rectal cancers is a surgical approach that combines TME with lateral lymph node dissection (LLND) without RT or CRT.

Eastern surgeons prefer LLND for sterilization of the lateral compartment, based on fears of CRT-associated late complications, such as radiation proctitis, pelvic fracture, and second carcinogenesis. The Japanese JCOG0212 trial [5] included patients with clinical stage II/III lower rectal cancer, and reported a local recurrence incidence of only 7% among patients who underwent TME with LLND, which is comparable to incidence rates reported in several Western studies. Moreover, the results of a comparative study between Japan and the Netherlands demonstrate that both TME + LLND and RT + TME resulted in good local control [6].

There are several drawbacks to LLND, including potentially increased incidences of sexual and urinary dysfunction after rectal cancer surgery. Additionally, the reported 7% incidence of pathological LN metastasis after LLND without CRT, among patients with clinical stage II/III cancer who were clinically negative for lateral pelvic LN metastasis, indicates that lateral lymphadenectomy is performed in over 90% of patients without histologically positive lateral pelvic lymph nodes (LPLN). All locally advanced rectal cancers below the peritoneal reflection are considered an indication for LLND according to the Japanese guidelines for lower rectal cancer treatment [7]. However, based on the low incidence of lymph node metastasis and the possibility of dysfunction due to autonomic nerve impairment, LPLN dissection is controversial, especially in patients with clinically negative lateral pelvic LNs. It would be ideal to perform LLND only when LPLN metastasis is highly suspected, to avoid overtreatment and morbidity. However, preoperative radiological examination remains insufficient for the detection of LPLN metastasis [8, 9].

A recently introduced concept is focused on the sentinel lymph node (SLN), i.e., the first lymph node to receive lymphatic flow from the tumor. SLN navigation surgery may lead to reasonable LN retrieval, and is clinically performed in breast cancer [10] and malignant melanoma [11]. More recently, the SLN concept has also been accepted for gastrointestinal cancer [12, 13]. We previously reported application of the SLN concept for detection of the lateral pelvic SLN (LPSN), and as an indication of LPLN dissection using a dye method with indocyanine green and a near-infrared camera system in open rectal surgery [14, 15].

Laparoscopic surgery for rectal cancer, including of the lower rectum, has recently gained wide acceptance. However, laparoscopic TME + LLND is technically challenging and can require a prolonged operative time. Notably, the development of optical instruments for laparoscopy has enabled easy diagnosis of perioperative lymph flow using ICG during laparoscopic surgery as well as open surgery. It is essential to evaluate whether the SN concept may be used to accurately diagnose LPLN involvement, and thus reduce the need for LLND in laparoscopic rectal cancer surgery.

In the present study, we aimed to clarify the possibility of LPSN navigation surgery using ICG method for detecting LPSN in laparoscopic rectal cancer surgery by reporting pathological LPSN and non-LPSN involvement status.

## Methods

### Patients

This study included the lower rectal cancer patients who were diagnosed with stage II or III preoperatively according to the UICC-TNM classificationl [16]. The LPLN involvement is possible confounding variable of SLN detection in the rectal cancer surgery. Pre-surgical examinations were done by colonoscopy, computed tomographic scanning, and magnetic resonance imaging, and revealed no enlargement in the lateral pelvic LNs (< 10 mm) in any of the included patients. In our database, 21 patients underwent curative laparoscopic surgery with SLN detectection followed by LLND between December 2016 and March 2019. All included patients did not received preoperative radiotherapy or chemotherapy. The number of samples required to verify whether the negative rate of lateral lymph node metastasis in this study was equivalent to the true negative rate of lymph node metastasis was calculated to be 20 cases. (From δ error 10%, confidence level 90%, population lymph node metastasis negative rate 93%).

This retrospective study was approved by the Human Ethics Review Committee of Osaka International Cancer Institute.

### SLN detection by ICG using the laparoscopic near-infrared camera system

**Fig. 1:**
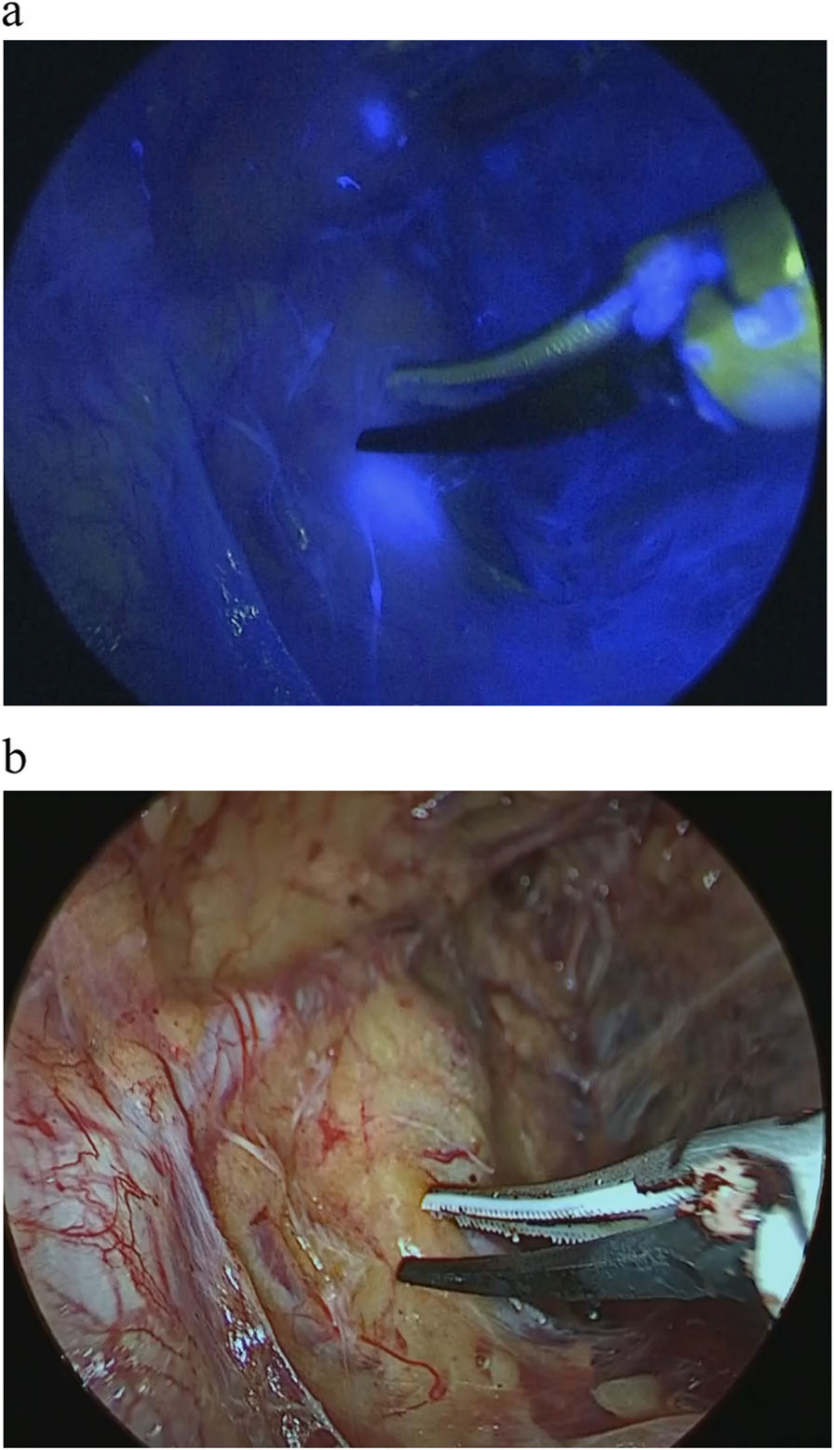
Detection of sentinel nodes around the internal iliac artery. **a** When using the laparoscopic near-infrared camera system, the lymph node that received indocyanine green appeared as a shining fluorescent spot. **b** The SLN that received ICG is not visibly stained blue when examined under white light

Before starting laparoscopic surgery, ICG dye (Diagnogreen; Dai-Ichi Pharm, Tokyo, Japan) was injected at submucosal layer on the anal side of the tumor, as previously described [14] For each patient, the total volume of injected dye was 1 mL (5 mg, 0.5% ICG). After completion of the laparoscopic TME, the SLN of bilateral lateral pelvic region was identified. The ureterohypogastric fascia containing the ureter and hypogastric nerve was exposed, and then the external iliac vessels were exposed and isolated from the peritoneal axis up to the iliac bifurcation. Next, the lateral vesical and obturator space were cleared, between the lateral aspect of the internal iliac vessels and the pelvic wall. Then, the laparoscopic near-infrared camera system (KARL STORZ GmbH & Co. KG, Tuttlingen, Germany) was used to observe the adipose tissue of lateral pelvic region. ICG fluorescence imaging was generated by the laser-free full high-definition camera system (IMAGE 1 SPIES™, KARL STORZ) prepared with a specific filter for exposure of NIR fluorescence and white light. Both visible and NIR excitation light were provided by the xenon-based light source (D-LIGHT P SCB, KARL STORZ), and the conversion between standard light and NIR was easily switched (Fig. 1a,b). This system enabled SLN identification in the lateral pelvic region. We defined all the LNs received ICG appearing shining fluorescent spot in the image as sentinel nodes. After LPSN identification and biopsy, all patients underwent laparoscopic bilateral LLND. The common-internal iliac vessels and the obturator nerve were completely cleared, from the lymphatic tissue down to the inferior vesical vessels and obturator fossa.

The time required for LPSN biopsy and the amount of bleeding during the procedure were calculated from the recorded surgical video and anesthesia chart.

### Division of the lateral pelvic region

**Fig. 2:**
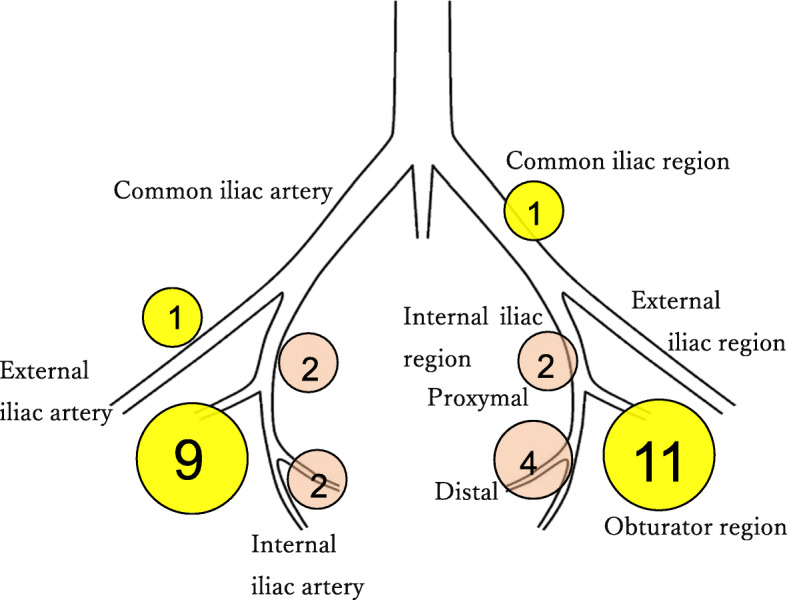
Diagram showing the division of the lateral pelvic region, and the SLN locations in 16 patients. The numeral indicates the number of patients with an SLN in this area. Some patients showed SLNs in multiple locations

We divided the lateral pelvic region whitch contains lateral lymph node into three regions according to the Japanese Classification of Colorectal Carcinoma: the common iliac region; the internal iliac region; and the obturator region along the obturator nerve, artery, and vein. (Fig. 2) [17].

### Endpoint and statistical analysis

The primary endpoint of this study is to show the negative predictive value and specificity of sentinel lymph node biopsy. Statistical analyses were performed using the SPSS version 24.0 software package. Data are presented as mean ± standard deviation. We analyzed the data using the chi-square test. We also used the Fisher’s exact test when the sample size was small. A *p* value of < 0.05 was considered to indicate a statistically significant difference.

## Results

### Patient characteristics

This study included 21 patients [11 men (52.4%) and 10 women (47.6%], with a median age of 68 years (range, 43–80 years). The mean tumor size was 45 mm (range, 10–80 mm). For all tumors, the lower anal edge was located at or below the peritoneal reflection. The mean distance from the anal verge to the lower edge of the tumor was 50 mm (range, 10–70 mm). Moderate differentiated adenocarcinoma was the most common in 14 cases. Well-differentiated adenocarcinoma and mucinous adenocarcinoma were 6 cases and 1 case, respectively. (Table 1).

**Table 1:** Patients’ baseline characteristics (*n* = 21)

Characteristic	Value
Sex, Male/Female	11/10
Age in years, median (range)	68 (43–80)
Tumor size in mm, median (range)	45 (10–80)
Distance from anal verge in mm, median (range)	50 (10–70)
Clinical T stage T1/2/3/4	0/2/15/2
Clinical N stage N0/1/2	11/8/2
UICC TNM stage II/III	11/10
Histology well/mod/muc	6/14/1

### Detection of lateral SLNs

The lymph vessels and LNs that contains ICG could be seen as sparkle fluorescent flow and spots in the image from the laparoscopic near-infrared camera system. We successfully identified the lateral SLNs in 16 (76.2%) of the 21 patients. The aggregate number of lateral SLNs was 32. The median number of lateral SLNs per patient was 2 (range, 1–15). Among the 16 patients with LPSN identification, the LPSNs were most commonly detected in the obturator region. Figure 2 shows the locations of the LPSNs identified in 16 patients. Successful lateral SLN identification was not influenced by the distance from anal verge, tumor size, histological grade, primary tumor factor, clinical and pathological upward LN metastasis status, pathological LPLN metastasis status, lymphatic invasion, or venous invasion (Table 2). The procedure time and bleeding volume during LPSN biopsy were 42 min (median, 25–68 min) and 0 g (median, 0–20 g) (recorded on the chart), respectively.

**Table 2:** Clinico-pathological characteristics and sentinel lymph node (SLN) detection

Clinico-pathological characteristic	SLN detected (***n*** = 16)	SLN not detected (***n*** = 5)	***p*** value
Distance from anal verge			
<50 mm	6	3	0.611
≥50 mm	10	2	
Tumor size			
<45 mm	4	4	0.047
≥45 mm	12	1	
Histology			
Well/mod	15	5	1.000
muc	1	0	
Pathological T stage			
T3/4	12	2	0.28
T1/2	4	3	
Clinical upward lymph node metastasis			
Negative	8	3	1.000
Positive	8	2	
Pathological upward lymph node metastasis			
Negative	8	3	1.000
Positive	8	2	
Pathological LPLN metastasis status			
Negative	15	4	0.429
Positive	1	1	
Lymphatic invasion			
No	15	3	0.128
Yes	1	2	
Venous invasion			
No	4	2	0.598
Yes	12	3	

### Correlation between SLNs and the non-sentinel lymph nodes

We performed hematoxylin and eosin (HE) staining to examine the metastatic status of SLNs and non-SLN lymph nodes. In cases where the lateral SLN was identified with the laparoscopic near-infrared camera system, we determined assessed the LPSN for metastasis using an intraoperative rapid diagnosis with HE staining. We additional determined whether the patients exhibited metastasis in the dissected non-SLN lateral lymph nodes by performing pathological diagnosis with HE staining postoperatively. LLND was performed in all 21 patients (16 patients with identified lateral SLNs, and 5 patients without SLN identification). The median number of dissected lateral lymph nodes was 15 (rages, 2–39). Of the five patients without LPSN identification, one patient exhibited lateral lymph node metastasis based on permanent HE staining, while the other four patients showed negative lateral LNs. Among the 16 patients with identified lateral SLNs, one exhibited metastasis in the SLN based on intraoperative HE staining. The dissected lateral non-SLN lymph nodes from this patient did not also show metastasis. Among the remaining 15 patients with no metastasis observed in the identified LPSN, all of their dissected lateral non-SLN lymph nodes were also metastasis free. (Table 3) Therefore, among patients with metastasis-negative lateral SLNs, the negative predictive value was 100% (15/15). Of the 19 patients with no metastasis in the LPSN and lateral non-SLN lymph nodes, 15 patients showed no metastasis in the identified LPSN. Therefore, specificity of SLN biopsy was 78.9% (15/19).

**Table 3:** Status of sentinel lymph nodes (SLNs) and non-SLN lymph nodes according to hematoxylin and eosin staining

SLN identification	Intra-operative SLN diagnosis	Diagnosis of non-SLN dissected lateral lymph nodes
Identified: *n* = 16	+ SLNs: *n* = 1	+ non-SLNs: *n* = 0
		− non-SLNs: *n* = 1
	− SLNs: *n* = 15	+ non-SLNs: *n* = 0
		− non-SLNs: *n* = 15
Not identified: *n* = 5	Not available: *n* = 5	+ non-SLNs: *n* = 1
		− non-SLNs: *n* = 4

### Short term outcomes and local recurrence

In our patients who underwent SLN biopsy followed by LLND, Grade 3–4 postoperative complications occurred in 5 (23.8%) patients. The most common grade 3 or 4 postoperative complication was perineal abscess after abdominal perineal resection (3 [14.3%] pateints). No anastomotic leakage was observed. The incidence of early urinary dysfunction (residual urine volume > 100 mL) occurred in 6 (28.6%) patients. Grade 3 urinary retention was observed in two patients (9.5%). No patients developed urinary incontinence.

One patient suffered local recurrence. The median length of follow-up for censored cases was 24.1 months (range, 16.8–44.4 months).

## Discussion

The presently investigated sentinel node technique is based on the theory that the tumor-bearing status of the SLN reflects the tumor status of the remaining nodes. Our results supported this theory, in that the absence of lateral SLN metastasis reflected an absence of metastases in the non-sentinel LNs at the time of laparoscopic surgery. However, this study is a feasibility study conducted with the minimum number of patients required. Our results don’t fulfill criteria for implementation into clinical practice avoiding LLND, but might show promising results that have to be confirmed by larger and properly-designed studies. To consider the future clinical application of SLN biopsy to prevent LLND, it is important that the diagnosis has a high negative-predictive value and high sppecificity. In our present study, the negative predictive value and the specificity was 100 and 78.9%, respectively. Thus, our results suggest the potential use of this SLN strategy to identify cases with non-metastatic LPLN, and to omit LLND in such cases, and thereby avoid both LLND-related surgical complications and radiation-induced adverse events. To further validate the clinical use of our presently described method, prospective studies of the SLN biopsy-based LLND strategy should be performed with large numbers of patients, and including examination of local control and survival. In addition, further research is needed to shorten the required time and improve the accuracy of SLN biopsy by the intraoperative rapid diagnosis with different methods such as molecular biological diagnosis.

In our cohort, the SLN was identified in about 75% of patients (16/21), indicating that this is a useful method that many people may benefit from. In a previous study of colorectal cancer, Cahill and colleagues [18] report the identification of SLNs using ICG dye in all cases. The SLN identification rate in our study was lower, although it was comparable to the identification rate during open surgery in our previous study. There are several possible reasons for the difference between our SLN identification rate and that reported by Cahill et al. First, we could not identify SLNs in all of our rectal cancer cases due to a loss of visibility in dense fat, and the rapid transit of the ICG in our dye-guided SLN method. A novel laparoscopic ICG fluorescence imaging system has been developed to overcome this limitation of ICG-guided SLN biopsy, and further technological developments are expected to improve SLN detection. Second, the lymphatic flow in the lower rectum is highly complex, moving in both an upstream direction and a lateral direction, and the distribution of this bidirectional lymph flow varies among different cases. Some patients may even lack the lateral pelvic lymph flow. Third, in some cases, ICG failed to pass through the lymphatic vessels. The group of five cases without SLN identification included one case with LPLN metastasis, suggesting the possibility that the presence of cancer cells may prevent ICG passage in the lymphatic vessels. These findings suggest that it may not be appropriate to employ the SLN biopsy-based treatment strategy in cases where preoperative imaging suggests a high likelihood of lateral lymph node metastasis.

In our cohort, two patients exhibited lateral lymph node metastasis: one with and one without SLN identification. This suggests that laparoscopic LLND should be performed unless SLN identification is successful and the biopsy is negative. Lateral lymph node metastasis was found in about 10% (2/21) of our included patients. This incidence of lymph node metastasis is comparable to those reported in the JCOG0212 trial, [5] in which all included patients were treated with TME + LLND. Our present results may indicate that the SLN biopsy strategy could have been used to efficiently determine which cases required lateral lymph node dissection.

It is important to know where and how to sample the sentinel lymph nodes. Many prior studies of rectal cancer have examined lymph flow in the lateral direction. The lymphatic pathways of the low rectum reportedly drain both cranially along the superior rectal vessels and laterally along the middle rectal vessels, [1, 4] and then to the internal iliac vessels [2, 3]. Additionally, we have mapping data from laparotomy surgery, indicating which lymph nodes are likely to be identified as SLNs using ICG. In our present laparoscopic surgery study, most SLNs were identified in the obturator region (283 as described in Japanese guidelines), followed by in the internal iliac region (263), which was consistent with previous data from open rectal cancer surgery [14]. Most of the LNs identified as SLNs were found in either the Internal iliac region or the obturator region. During SLN biopsy, surgeons should pay particular attention to these areas.

Sampling operations raise concerns about whether destruction of lymph nodes and lymph flow will lead to an outflow of cancer cells. However, autonomous nerve-preserving LLND, in which the lymph nodes are excised from each basin, is performed after TME rather than along with excision of the main tumor. In our performance of SLN biopsy, we exposed the fascia along the autonomic nerve system, internal iliac vessels and urinary vessels, and pelvic side wall to expose preserved organ—and then only the shiny lymph nodes seen through there were identified as sentinel lymph nodes—and, importantly, the lymph nodes were not destroyed. Notably, sentinel lymph node sampling in other cancer types has not been reported to cause oncological spread.

In this study, sentinel lymph nodes were defined as the only lymph nodes that glowed in vivo. However, in ex vivo observation, additional lymph nodes that are not visible through the fascia may also emit excitation light in ICG. The relatively small number of SLNs identified per case (median of two) was likely due to the identification of sentinel lymph nodes in vivo. One limitation of our study is that we lacked data regarding potentially shiny LNs that could have been identified ex vivo. However, to apply the SLN theory to reduce the performance of LLND in rectal cancer surgery, we believe that the data regarding SLN identification in vivo is relevant—not the ex vivo identification.

Another limitation of this study is that it was a retrospective cohort study of patients who underwent intraoperative SLN biopsy and dissection of lateral non-SLN lymph nodes. There may be confounders influencing the concordance between SLN status and non-SLN status, potentially including statistical error from the relatively small number of patients, and sampling error due to the complexity of lateral pelvic anatomy. However, in our study, SLN biopsy was performed by only one surgeon who has extensive experience with laparoscopic LLND, which likely reduced sampling error. In this study, all patients underwent LLND after LPSN biopsy, so it was not possible to compare sexual or urinary function between patients who underwent LPSN biopsy alone and those who underwent LLND. However, the LPSN biopsy procedure showed more than an hour less surgery time [19] and less bleeding than the reported LLND procedure.

## Conclusions

In conclusion, although further study is necessary, our present results suggest that the laparoscopic ICG-guided SLN strategy is a potentially harmless and time-saving method that could lead to the omission of laparoscopic LLND in a considerable number of patients with lower rectal cancer.

## Supplementary Information

**Additional file 1.**

## Data Availability

The data has been included as digital supplementary material.

## Abbreviations

TME: total mesorectal excision
LLND: lateral lymph node dissection
RT: radiotherapy
LPLN: lateral pelvic lymph nodes
SLN: sentinel lymph node
CRT: chemoradiotherapy
LPSN: lateral pelvic sentinel lymph nodde
HE: hematoxylin and eosin

## References

[1] Gerota D (1895). Die lymphgefasse des rectums und des anus. Arch Anat Physiol.

[2] Senba Y (1927). An anatomical study of the lymphatic system of the rectum. J Hukuoka Med Coll.

[3] Villemin F, Huard P, Montagne M (1925). Recherches anatomiques sur les lymphatiques du rectum et de l’anus. Rev Chir.

[4] Poirier P, Cuneo B, Delamere G (1904). The lymphatics.

[5] Fujita S, Mizusawa J, Kanemitsu Y, Ito M, Kinugasa Y, Komori K, Ohue M, Ota M, Akazai Y, Shiozawa M, Yamaguchi T, Bandou H, Katsumata K, Murata K, Akagi Y, Takiguchi N, Saida Y, Nakamura K, Fukuda H, Akasu T, Moriya Y, Colorectal Cancer Study Group of Japan Clinical Oncology Group (2017). Mesorectal excision with or without lateral lymph node dissection for clinical stage II/III lower rectal Cancer (JCOG0212): a multicenter, randomized controlled, noninferiority trial. Ann Surg.

[6] Kusters M, van de Velde CJ, Beets-Tan RG, Akasu T, Fujita S, Yamamoto S, Moriya Y (2009). Patterns of local recurrence in rectal cancer: a single-center experience. Ann Surg Oncol.

[7] Hashiguchi Y, Muro K, Saito Y, Ito Y, Ajioka Y, Hamaguchi T, Hasegawa K, Hotta K, Ishida H, Ishiguro M (2020). Japanese Society for Cancer of the Colon and Rectum (JSCCR) guidelines 2019 for the treatment of colorectal cancer. Int J Clin Oncol.

[8] Amano K, Fukuchi M, Kumamoto K, Hatano S, Ohno H, Osada H, Ishibashi K, Ishida H (2020). Pre-operative evaluation of lateral pelvic lymph node metastasis in lower rectal Cancer: comparison of three different imaging modalities. J Anus Rectum Colon.

[9] Kijima S, Sasaki T, Nagata K, Utano K, Lefor AT, Sugimoto H (2014). Preoperative evaluation of colorectal cancer using CT colonography, MRI, and PET/CT. World J Gastroenterol.

[10] Lyman GH, Somerfield MR, Bosserman LD, Perkins CL, Weaver DL, Giuliano AE (2017). Sentinel lymph node biopsy for patients with early-stage breast Cancer: American Society of Clinical Oncology clinical practice guideline update. Journal of clinical oncology: official journal of the American Society of Clinical Oncology.

[11] Wong SL, Faries MB, Kennedy EB, Agarwala SS, Akhurst TJ, Ariyan C, Balch CM, Berman BS, Cochran A, Delman KA, Gorman M, Kirkwood JM, Moncrieff MD, Zager JS, Lyman GH (2018). Sentinel lymph node biopsy and Management of Regional Lymph Nodes in melanoma: American Society of Clinical Oncology and Society of Surgical Oncology clinical practice guideline update. Ann Surg Oncol.

[12] Shimada A, Takeuchi H, Nishi T, Mayanagi S, Fukuda K, Suda K, et al. Utility of the one-step nucleic acid amplification assay in sentinel node mapping for early gastric cancer patients. Gastric Cancer. 2019. 10.1007/s10120-019-01016-9 31667687

[13] Isozaki H, Matsumoto S, Murakami S (2019). Survival outcomes after sentinel node navigation surgery for early gastric cancer. Ann Gastroenterol Surg.

[14] Noura S, Ohue M, Seki Y, Tanaka K, Motoori M, Kishi K, Miyashiro I, Ohigashi H, Yano M, Ishikawa O, Miyamoto Y (2010). Feasibility of a lateral region sentinel node biopsy of lower rectal cancer guided by indocyanine green using a near-infrared camera system. Ann Surg Oncol.

[15] Noura S, Ohue M, Seki Y, Yamamoto T, Idota A, Fujii J, Yamasaki T, Nakajima H, Murata K, Kameyama M, Yamada T, Miyashiro I, Ohigashi H, Yano M, Ishikawa O, Imaoka S (2008). Evaluation of the lateral sentinel node by indocyanine green for rectal cancer based on micrometastasis determined by reverse transcriptase-polymerase chain reaction. Oncol Rep.

[16] Brierley JD, Gospodarowicz MK, Wittekind C. TNM classification of malignant tumours: John Wiley & Sons; 2017.

[17] Ogura A, Konishi T, Cunningham C, Garcia-Aguilar J, Iversen H, Toda S, Lee IK, Lee HX, Uehara K, Lee P, Putter H, van de Velde CJH, Beets GL, Rutten HJT, Kusters M, on behalf of the Lateral Node Study Consortium (2019). Neoadjuvant (chemo)radiotherapy with Total Mesorectal excision only is not sufficient to prevent lateral local recurrence in enlarged nodes: results of the multicenter lateral node study of patients with low cT3/4 rectal Cancer. Journal of clinical oncology: official journal of the American Society of Clinical Oncology.

[18] Cahill RA, Anderson M, Wang LM, Lindsey I, Cunningham C, Mortensen NJ (2012). Near-infrared (NIR) laparoscopy for intraoperative lymphatic road-mapping and sentinel node identification during definitive surgical resection of early-stage colorectal neoplasia. Surg Endosc.

[19] Morohashi H, Sakamoto Y, Miura T, Kagiya T, Ogasawara K, Takahashi Y, et al. Short-term outcomes of robotic-assisted laparoscopic versus laparoscopic lateral lymph node dissection for advanced lower rectal cancer. Surg Endosc. 2020. 10.1007/s00464-020-07979-6. 10.1007/s00464-020-07979-6 PMC8346387 33006031

